# Divergent responses of plant lignin and microbial necromass to the contribution of soil organic carbon under organic and chemical fertilization

**DOI:** 10.3389/fmicb.2025.1586791

**Published:** 2025-05-16

**Authors:** Xingping Chang, Zhanjiang Pei, Xiaofei Wang, Hao Wang, Jie Mu, Yingjun Ma, Mingye Zhang, Keqiang Zhang, Lianzhu Du

**Affiliations:** ^1^Agro-Environmental Protection Institute, Ministry of Agriculture and Rural Affairs, Tianjin, China; ^2^Heilongjiang Academy of Black Soil Conservation & Utilization, Heilongjiang Academy of Agricultural Sciences, Harbin, China; ^3^Institute of Plant Nutrition, Agricultural Resources and Environmental Sciences, Henan Academy of Agricultural Sciences, Zhengzhou, China; ^4^Key Laboratory of Low-carbon Green Agriculture in North China, Ministry of Agriculture and Rural Affairs, Beijing, China

**Keywords:** soil organic carbon, plant-derived carbon, microbial-derived carbon, microbial CAZyme families, cattle slurry

## Abstract

**Introduction:**

Long-term fertilization strongly influences the formation, turnover and stabilization of soil organic carbon (SOC) in croplands. The underlying mechanism by which chemical fertilizer (CF) and cattle slurry (CS) application influence the dynamics of plant- and microbial-derived carbon (C) remains elusive.

**Methods:**

Lignin phenols and amino sugars as well as microbial carbohydrate-active enzymes (CAZymes) were analyzed in a 10-year field experiment.

**Results:**

CF and CS increased the content of SOC by 26.9% and 88.0% compared with the SOC content of an abandoned land, respectively. CS application increased lignin phenols content by 4.28 times compared with CF application owing to slower oxidative degradation and higher plant inputs. Microbial necromass C (MNC) contents increased by 34.7% in line with an increase in biomass, but its proportion to SOC did not change. In terms of microbial community, the application of CF and CS altered the distribution and structure of microbial community. The dominant bacterial phyla shifted from oligotrophic Actinomycetota to eutrophic Pseudomonadota with change in the application from CF to CS. CS application increased CAZyme genes associated with plant- and bacterial-derived fractions decomposition, suggesting higher degradation potential of plant and bacterial biomass by microorganisms. Availability of substrates and microbial community are important factors affecting SOC accumulation in different fertilization treatments.

**Discussion:**

CS application considerably promoted the accumulation of SOC, mainly via the promotion of plant-derived C. Alterations in microbial communities and CAZyme genes could affect microbial metabolism via “microbial carbon pump”, thereby facilitating SOC formation and accumulation.

## 1 Introduction

Soil, as the largest terrestrial carbon (C) sink, contains 2–3 times more C than atmosphere and 3–4 times more than vegetation (Friedlingstein et al., 2020; Jackson et al., 2017). Consequently, a slight variation in soil organic carbon (SOC) sink may greatly affect global climate. The formation, turnover and stabilization of SOC are critical processes for C cycling in the terrestrial biosphere, and the accrual of SOC in croplands has a considerable impact on global C storage (Liu et al., 2022; Yang et al., 2023). In traditional perspective, plant residues were considered as the primary contributor to stable C sink, such as lignin, owing to their recalcitrant chemical structure (Liang et al., 2019). Growing studies agree with the view that microbial necromass is a vital source of the stable C sink, which can be transformed from plant residues, and can constitute more than half of SOC in agricultural ecosystems (Camenzind et al., 2023; Chen G. P. et al., 2020). The “microbial carbon pump” framework underlines that microbial metabolism plays a vital role in SOC formation and turnover (Liang et al., 2017; Zhu et al., 2020). Plant components can serve as energy resources for soil microorganisms growth and subsequently be converted to microbial fractions through microbial metabolic pathways: *ex vivo* modification and *in vivo* turnover (Liang et al., 2017; Sokol et al., 2019; Whalen et al., 2022). Microbial residues can bind to soil mineral particles and be physically protected by soil aggregates, which makes them more resistant to be broken down than plant residues (Chen G. P. et al., 2020). Plant-and microbial-derived C are normally quantified by biomarkers, including lignin phenols and amino sugars (Liang et al., 2019; Ma et al., 2018; Su et al., 2023). Therefore, elucidating plant-and microbial-derived C dynamics and their impacts on accumulation of SOC in agricultural ecosystem is essential.

Soil microorganisms participate in the breakdown of plant components and synthesis of microbial compounds, thus affecting terrestrial C cycling processes (Liang, 2020; López-Mondéjar et al., 2018). Recently, carbohydrate-active enzymes (CAZymes) are used to assess microbial metabolic pathways involved in a range of biomolecules (Ren C. J. et al., 2021; Zhang C. et al., 2021). Variations of CAZymes species and abundance can characterize the shift in the ability of soil microbial communities to degrade various organic compounds (Yan et al., 2022). Specific glycoside hydrolases (GHs) and auxiliary enzymes (AAs) have been proven to be responsible for plant necromass breakdown (Bomble et al., 2017; Llado et al., 2019). For example, the cellulolytic enzyme from GH6 is related to cellulose degradation, and peroxidase (POD) from several AAs is responsible for lignin breakdown (López-Mondéjar et al., 2020; Ren C. J. et al., 2021). Chitinases and lysozymes from some GHs are associated with fungal and bacterial residue decomposition (Zifcáková et al., 2017). Soil substrates and environmental variables strongly influence CAZyme activities owing to their effects on microbial communities (Ren C. J. et al., 2021). Cropland management practices, such as fertilization, cause large impacts on soil substrate and environmental factors, further altering microbial utilization of C sources and thus affecting CAZyme genes response for the breakdown of plant and microbial residues (Hu et al., 2020; Liu et al., 2019). Clarifying the shifts of CAZyme gene families and their relevance with organic components decomposition is vital to understand the microbial-mediated SOC dynamics in croplands.

Extensive researches have shown that fertilization is an effective management practice that facilitates the maintenance of soil fertility, improves crop yields, as well as promotes C accumulation in agricultural ecosystem (Ren F. L. et al., 2021; Xiong et al., 2023; Yan et al., 2007). Chemical fertilizer (CF) application can boost crop production, increasing plant inputs and root exudates into the soil and thus substantially influencing C dynamics (Kicklighter et al., 2019). Cattle slurry (CS) application is regarded to be profitable for soil fertility and crop growth due to its abundance of nutrient substrates and organic components (Chen et al., 2024; Lu et al., 2012). However, exogenous C sources imported by CS application and organic components produced by crop, along with C loss from degradation, make the SOC turnover in response to CS application unclear (Peltre et al., 2017; Yang et al., 2015). This study was based on the long-term fertilization experiments in Harbin City and aimed to (1) compare the impacts of CF and CS application on microbial- and plant-derived C and their contributions to SOC, (2) evaluate the distribution and abundance of CAZyme genes involved in microbial and plant components decomposition under different fertilizer applications, and (3) elucidate the main factors driving SOC variations across different fertilizer applications. We hypothesized that (1) long-term CF and CS applications would promote the accumulation of microbial- and plant-derived C, and the increase in SOC under CS application would mainly due to the enhanced contribution of MNC to SOC, (2) CS application would shift the distribution of soil microorganisms and increase the abundance of CAZyme genes responsible for the breakdown of plant and microbial compounds more than CF application, and (3) the bacterial community may be the main factor regulating SOC accumulation owing to its rapid growth in rich-nutrient soil.

## 2 Materials and methods

### 2.1 Research site and sample collection

The long-term fertilization study started in 2013 and was located in Harbin City, Heilongjiang Province (45°24′N, 126°22′E), where the soil was categorized as Mollisol. The climate of the study area is characterized as temperate monsoon with a mean annual temperature of 4.2°C and a mean annual precipitation of 523 mm. The planting pattern involved planting maize crop once in a year, with stems and leaves cleared from the field after harvest and in the following spring stubble ploughed into the soil. In the current study, three treatments, each with three replicates, were arranged. The treatments consisted of (1) CF treatment: CF of 245 kg N ha^−1^, 96 kg P_2_O_5_ ha^−1^, and 60 kg K_2_O ha^−1^, (2) CS treatment: cattle slurry of 135 m^3^ ha^−1^, and an abandoned field (CK) that had ceased maize cultivation and fertilization for several years. Due to field and management constraints, a maize-cultivated but unfertilized control treatment was not available. Therefore, CK serves as a background reference reflecting the cessation of fertilization and cultivation.

In April 2023, soil samples were collected from the 0–20 cm topsoil layer of each plot and homogenized. Visible gravel and plant litter were manually removed. After passing through a 2 mm sieve, the soil samples were divided into three sub-samples: one part was for the determination of soil dissolved organic carbon (DOC) and microbial biomass carbon (MBC), which was stored at 4°C; the second part was for the analysis of soil microbial community and stored at −80°C immediately, and the third part was freeze-dried for the measurements of soil physical and chemical properties, amino sugars, lignin phenols, and enzyme activities.

### 2.2 Soil physical and chemical property analysis

SOC was quantified using a total organic carbon (TOC) analyzer (Vario TOC Select; Elementar, Germany). MBC was evaluated using the chloroform fumigation method, with a factor of 0.45 applied for calculation. DOC was determined using the TOC analyzer after extraction from the suspension of a 1:5 soil to water ratio. Soil total nitrogen (TN), nitrate nitrogen (NN), ammonium nitrogen (AN), total phosphorus (TP), soil available phosphorus (AP), and soil pH were analyzed via standard analytical processes, with details available in the Supplementary material.

Four soil extracellular enzymes related to C cycling, including peroxidase (POD), polyphenol oxidase (PPO), β-1,4-glucosidase (BG), and β-1,4-d-cellobiohydrolase (CBH) enzymes, were measured following the methods described in the Supplementary material. POD and PPO are oxidative enzymes with the ability to break down resistant organic compounds, such as lignin (Sinsabaugh, 2010). BG and CBH are hydrolytic enzymes capable of decomposing polysaccharides into monosaccharides, including cellulose and hemicellulose (Chen J. et al., 2020).

### 2.3 Lignin phenol analysis

Lignin phenols were quantified by the CuO oxidation method (Otto et al., 2005). Briefly, 0.5 g of soil, CuO, ammonium iron sulfate, and NaOH solution were mixed in Teflon vessels. After heating at 150°C, the solution was acidified to pH 1 and centrifuged. The supernatant was extracted with addition of Ethyl acetate, and the mixture was blown to near dryness. Ethylvanillin was used as the internal standard, and a methanol and dichloromethane (V:V = 1:1) solution was added to dissolve the mixture, then dried under nitrogen. The mixture was derivatized with methoxyamine hydrochloride and MATFA at 37°C. The derivatized compounds were analyzed using an Agilent 5,977 GC–MS equipped with a DB-35MS column (30 m × 0.25 mm × 0.25 μm).

Total lignin phenol (TLP) contents were the sum of vanillyls (V), syringyls (S), and cinnamyls (C). Vanillin, acetovanillone, and vanillic acid are included in V-typer phenols, syringaldehyde, acetosyringone, and syringic acid in S-typer phenols and p-coumaric and ferulic acid in C-typer phenols. Additionally, the S/V and C/V ratios were applied for the assessment of lignin biotransformation, and the acid-to-aldehyde ratios of vanillyls (Ad/Al) v and syringyls (Ad/Al) s were applied to evaluate the degree of lignin decomposition.

### 2.4 Amino sugar analysis

Amino sugars were extracted according to the method of Zhang and Amelung (1996). Soil samples were hydrolyzed, purified and derivatized, and the products were determined. Briefly, soil samples were hydrolyzed at 105°C, and then inositol was added as an internal standard. Subsequently, the hydrolysate was rotary evaporated, dissolved and adjusted pH to remove acids. In order to remove the salts, the residue was freeze-dried, dissolved in methanol, and freeze-dried again. After the addition of derivatization reagent to freeze-dried residue, the mixture was further acetylated with acetic anhydride at 80°C. Amino sugar derivatives were subsequently cleaned with HCl and water, dried under nitrogen and dissolved in hexane and ethyl acetate solution. Finally, the derivatized compounds were measured on a Thermo Scientific Trace 1,300 equipped with a TG-5SILMS column (30 m × 0.25 mm × 0.25 μm) and a flame ionization detector (FID).

Total amino sugar (TAS) contents were determined as the sum of glucosamine (GluN), galactosamine (GalN), and muramic acid (MurA). Microbial necromass C (MNC) contains fungal necromass C (FNC) and bacterial necromass C (BNC). FNC and BNC was calculated via GluN and MurA. The calculations were carried out using Equations 1–3:


(1) BNC=MurA×45

(2) $$\mathrm{FNC}=(\frac{\text{GluN}}{179.2}-2\times \frac{\text{MurA}}{251.2})\times 179.2\times 9$$

(3) MNC=BNC+FNC

Where 45 and 9 are the factors for converting MurA and GluN into BNC and FNC, respectively. 179.2 and 251.2 are the molecular weight of GluN and MurA, respectively.

### 2.5 Phospholipid fatty acid analysis

Phospholipid fatty acids (PLFAs) were employed for quantification of soil microbial biomass and were measured according to Frostegard and Baath (1996). In brief, freeze-dried soil samples were mixed with chloroform–methanol–citrate (V:V:V = 2:1:0.8) buffer and the solid-phase extraction column was used for separation of phospholipids. After phospholipids methylation, PLFAs methyl esters were quantified on an Agilent 7890B GC equipped with an FID and a 19091B-102 column (25 m × 0.2 mm × 0.33 μm). Specific PLFA peaks were identified using MIDI Sherlock Microbial Identification System (MIDI Inc., Newark, Delaware, USA).

The specific PLFAs selected as representative markers for various groups of microorganisms are shown in Supplementary Table S1. Total PLFAs were quantified by the summation of bacteria PLFAs, fungal PLFAs, and unspecified PLFAs, and the bacterial PLFAs consisted of gram-positive (G^+^) and gram-negative (G^−^) bacterial PLFAs. The ratios of G^+^/G^−^ bacterial PLFAs (G^+^/G^−^) and fungal/bacterial PLFAs (F/B) were applied to analyze microbial community composition.

### 2.6 DNA extraction, sequencing, and metagenomic analysis

Soil DNA was extracted using an E.Z.N.A.™ Soil DNA Kit (Omega Bio-Tek, Norcross, Georgia, USA). After examination of DNA purity and integrity and construction of paired-end libraries, the metagenomic sequencing was conducted on an Illumina Hiseq 2000 by Majorbio Biopharm Technology (Shanghai, China). The sequences have been uploaded to the National Center for Biotechnology Information, and RPJNA1166725 is the BioProject accession number. The metagenomic data was assembled with MEGAHIT (version 1.1.2), and then the nonredundant gene catalog was constructed with selection of contigs with overlap length over 300 bp for open reading frame prediction. The gene catalog was annotated according to the NR database^1^ to obtain microbial annotation and taxonomic information and the CAZyme database^2^ to identify specific functional gene families involved in organic compounds degradation. The reads per kilobase million (RPKM) was used to calculate the normalized abundance value. The detailed information of genes encoding microbial- and plant-derived component decomposition is shown in Supplementary Table S2.

### 2.7 Statistical analysis

Data normality was examined via the Shapiro–Wilk test. Significant differences of parameters among different fertilizer treatments were compared using one-way ANOVA based on the Duncan’s test (*p* < 0.05). Principal Coordinate Analysis (PCoA) was employed to analyze the similarity in the composition of soil bacterial and fungal community under different fertilization based on Bray–Curtis distance, and the significance of dissimilarity was tested by ANOSIM. Mantel test analysis was applied to assess the correction between CAZyme genes associated with the breakdown of microbial and plant compounds, soil enzyme activities and microbial community properties. Pearson correlation analysis was performed to explore the relationship between MNC (FNC and BNC), lignin phenols and soil environmental factors. Random forest analysis was used to assess the impacts of environmental variables on SOC, MNC, and lignin phenols. The percentage increase of the mean squared error (%IncMSE) was used to rank the relative importance of the environmental variables. All statistical analyses were performed in R software (R 4.3.3).

## 3 Results

### 3.1 SOC content and physical and chemical properties

CF and CS application led to 26.9 and 88.0% increase in SOC content compared with CK, respectively (Table 1). The enhancement effect was more pronounced in CS treatment. Similarly, the DOC contents increased by 53.8 and 221.0%, respectively. The MBC content reduced by 57.6% in CF treatment and enhanced by 233.8% in CS treatment. In addition, the amounts of TN, TP, and AP showed increasing trends, and CS application was more conducive to the improvement of soil nutrients.

**Table 1:** Soil physical and chemical properties in different treatments.

Properties	CK	CF	CS
SOC (g kg^−1^)	15.5 ± 0.7 c	19.4 ± 0.9 b	29.9 ± 2.5 a
DOC (mg kg^−1^)	41.5 ± 5.4 b	59.3 ± 8.1 b	175.6 ± 18.9 a
MBC (mg kg^−1^)	178.0 ± 22.5 b	75.4 ± 7.6 c	594.0 ± 67.3 a
pH	7.91 ± 0.04 a	5.47 ± 0.07 c	7.14 ± 0.16 b
TN (g kg^−1^)	1.29 ± 0.07 b	1.64 ± 0.02 b	3.02 ± 0.30 a
NN (mg kg^−1^)	9.82 ± 0.97 b	22.86 ± 1.26 a	38.07 ± 2.80 a
AN (mg kg^−1^)	0.58 ± 0.07 a	0.22 ± 0.02 b	0.48 ± 0.03 a
MBN (mg kg^−1^)	1.57 ± 0.17 b	2.23 ± 0.17 b	5.75 ± 0.31 a
C/N	12.0 ± 0.2 a	11.8 ± 0.4 a	10.0 ± 0.5 a
TP (g kg^−1^)	0.48 ± 0.02 b	0.55 ± 0.04 b	1.13 ± 0.05 a
AP (mg kg^−1^)	48.0 ± 1.8 b	55.2 ± 3.6 b	114.4 ± 4.3 a
PPO (μmol g^−1^ h^−1^)	0.32 ± 0.02 b	0.28 ± 0.02 b	0.51 ± 0.08 a
POD (μmol g^−1^ h^−1^)	1.20 ± 0.03 c	1.57 ± 0.02 a	1.33 ± 0.07 b
BG (μmol g^−1^ h^−1^)	1.17 ± 0.11 c	1.54 ± 0.05 b	2.05 ± 0.06 a
CBH (μmol g^−1^ h^−1^)	0.09 ± 0.01 b	0.14 ± 0.01 a	0.16 ± 0.01 a

SOC, soil organic carbon; DOC, dissolved organic carbon; MBC, microbial biomass carbon; TN, total nitrogen; NN, nitrate nitrogen; AN, ammonium nitrogen; MBN, microbial biomass nitrogen; C/N, the ratio of soil organic carbon to total nitrogen; TP, total phosphorus; AP, available phosphorus; PPO, polyphenol oxidase; POD, peroxidase; BG, β-1,4-glucosidase; CBH, β-1,4-D-cellobiohydrolases. Different letters indicate significant differences among different treatments (*p* < 0.05, Duncan’s test).

### 3.2 Amino sugars and microbial necromass in soil

**Figure 1:**
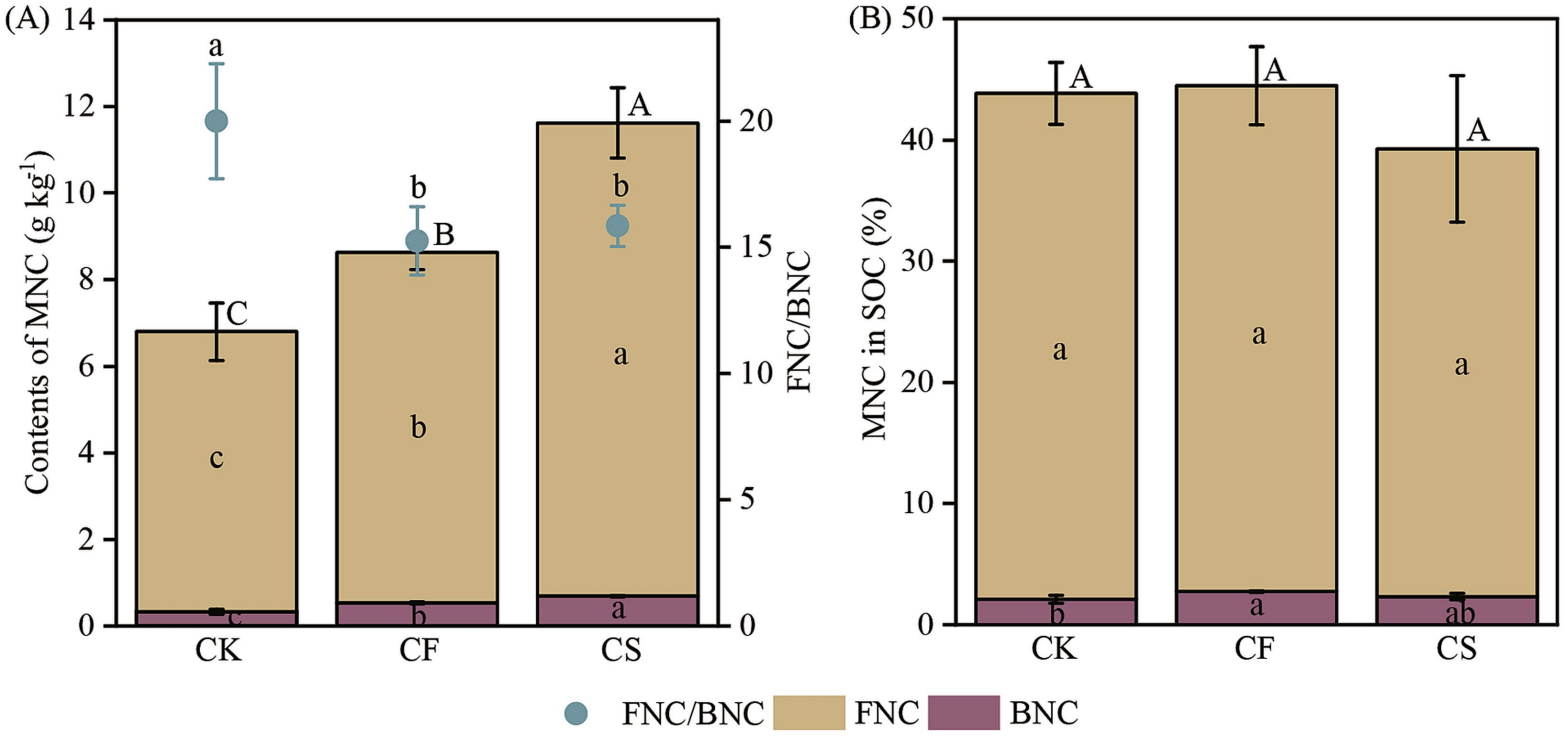
Contents of microbial necromass C and the ratio of fungal to bacterial necromass C **(A)**, and their contributions to SOC **(B)** in different treatments. Error bars represent standard errors of the means (*n* = 3). Different lowercase letters indicate significant differences in fungal and bacterial necromass C, and different uppercase letters indicate significant differences in total microbial necromass C among the treatments (*p* < 0.05, Duncan’s test).

The TAS concentration in different treatments were in the range of 0.97–2.05 g kg^−1^. The TAS contents in CF and CS treatments were 1.25–1.77 times larger than those in CK treatment (Supplementary Figure S1), with CS treatment having the highest content of TAS. GluN, GalN, and MurA showed a similar trend. The content of MNC ranged from 6.27 to 12.5 g kg^−1^ (Figure 1A). Compared with CF application, CS application increased FNC by 35.0% and BNC by 29.4%, resulting in a 34.7% rise in the MNC content (*p* < 0.05, Figure 1A). However, the contribution of MNC to SOC and FNC/BNC ratios showed no difference between the two fertilization treatments (*p* > 0.05, Figure 1).

### 3.3 Soil lignin phenols

**Figure 2:**
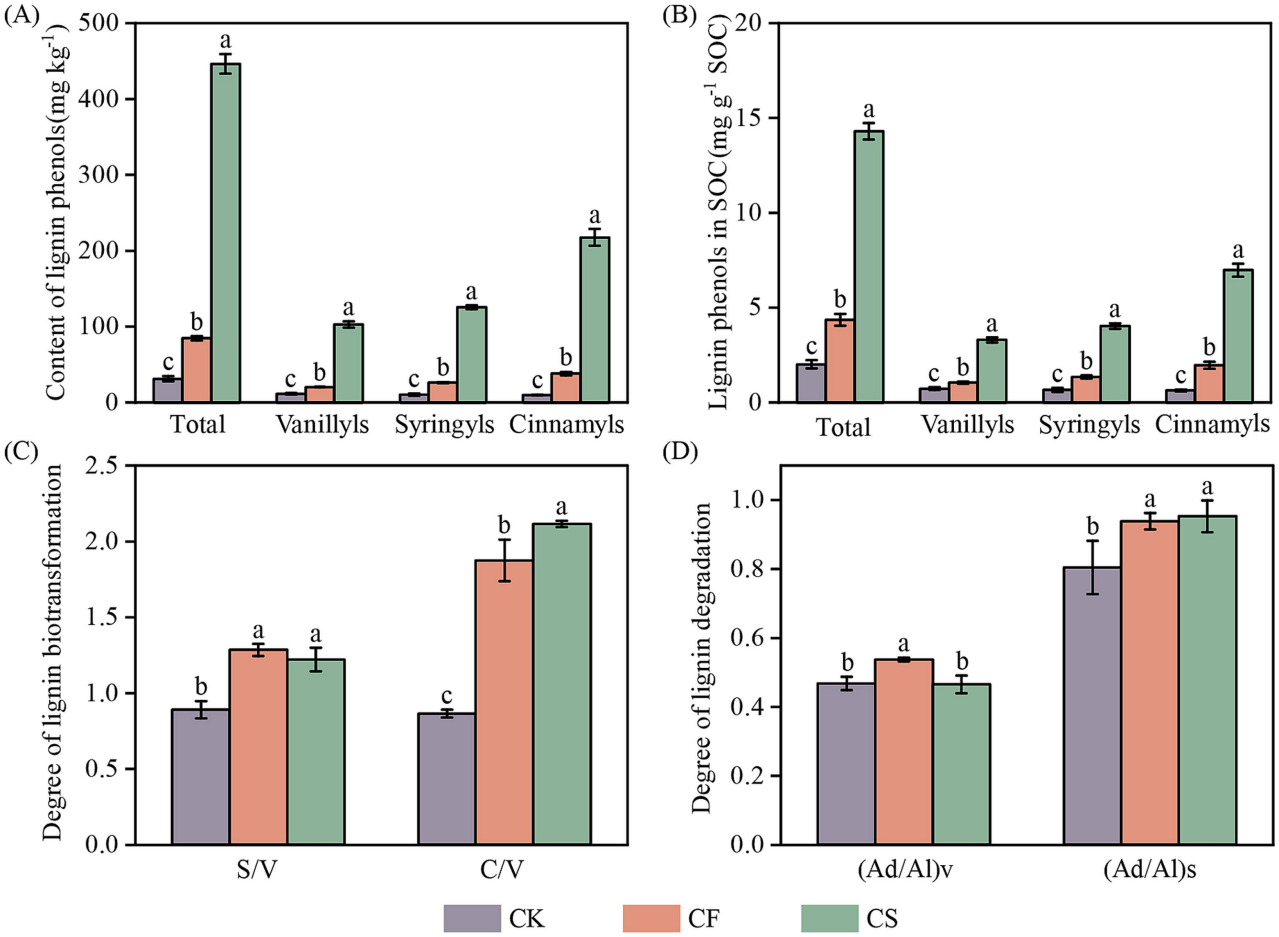
Contents of total and individual lignin phenols **(A)**, and their contributions to SOC **(B)**, the degree of lignin biotransformation **(C)** and degradation **(D)** in different treatments. The degree of lignin biotransformation was represented by the ratio of syringyl-type to vanillyl-type monomer (S/V) and cinnamyl-type to vanillyl-type monomer (C/V). The degree of lignin degradation was represented by acid/aldehyde ratio of vanillyl-type monomer (Ad/Al)v and acid/aldehyde ratio of syringyl-type monomer (Ad/Al)s. Error bars represent standard errors of the means (*n* = 3). Different letters indicate significant differences in total and individual lignin phenols, the degree of lignin biotransformation and degradation among the treatments (*p* < 0.05, Duncan’s test).

The contents of TLP ranged from 27.9 to 459 mg kg^−1^. The CS treatment showed the highest content, which was 4.28 times greater than CF treatment. CS treatment demonstrated a notable increase in the contribution of V-, S-, and C-type phenols to SOC compared with CF treatment (*p* < 0.05, Figures 2A,B). C-type phenols were predominant in lignin phenols, accounting for 43.6–49.9%, followed by S-type phenols (26.8–30.9%) and V-type phenols (22.8–25.1%) in CF and CS treatments. Fertilization affected lignin biotransformation. There was a 1.13 times greater ratio of C/V in CS application compared with CF treatment, while the (Ad/Al)v ratio reduced by 13.4% (Figures 2C,D). No difference was found in the ratios of S/V and (Ad/Al)s between the two treatments.

### 3.4 Characteristics of soil microbial community

**Figure 3:**
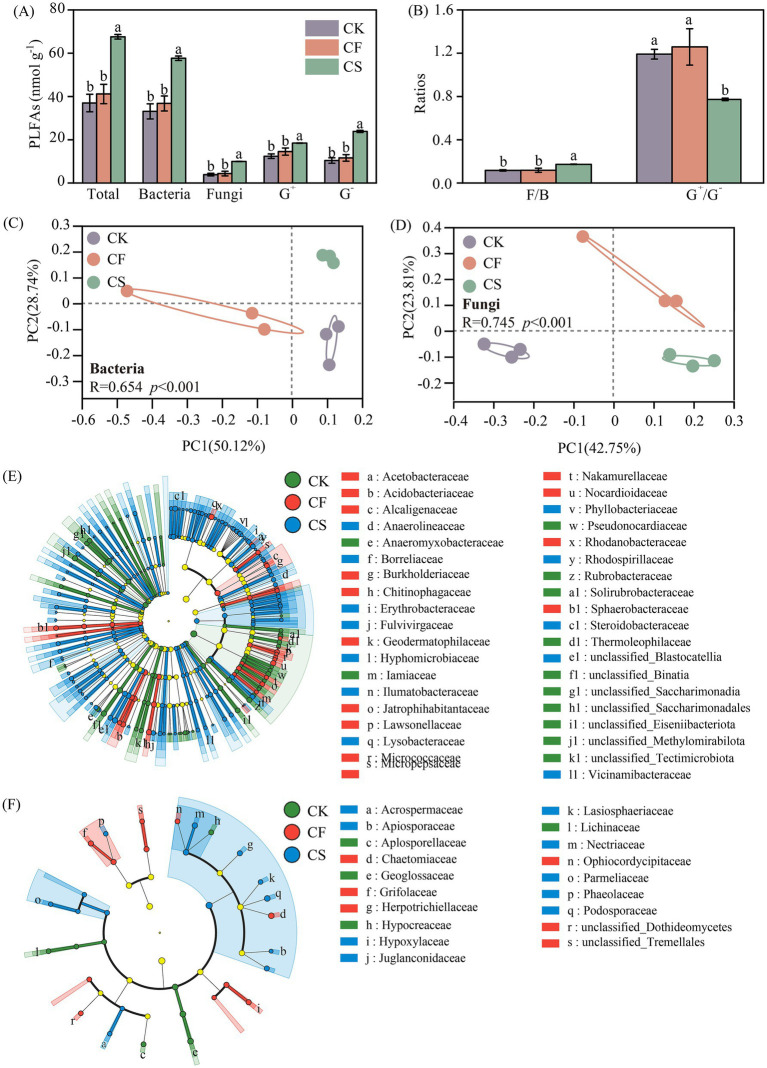
Contents of total, bacterial, fungal, gram-positive (G^+^) bacterial and gram-negative (G^−^) bacterial PLFAs **(A)**, and the ratio of fungal to bacterial PLFAs and gram-positive to gram-negative bacterial PLFAs **(B)** in different treatments. Difference of bacterial **(C)** and fungal **(D)** community structure based on the principal coordinate analysis (PCoA) and biomarker taxa compositions at the family level (LDA > 3, *p* < 0.05) of bacterial **(E)** and fungal **(F)** community. Error bars represent standard errors of the means (*n* = 3). Different letters indicate significant differences in total and individual PLFAs among the treatments (*p* < 0.05, Duncan’s test).

CS application considerably enhanced the total microbial biomass compared with CF application. Similar trends were observed in the biomass of G^+^ bacteria, G^−^ bacteria, bacteria, and fungi (Figure 3A). Compared with CF treatment, CS application increased the total microbial biomass by 64.2%, of which bacterial and fungal biomasses increased by 56.7 and 127%, respectively. The increase in bacterial biomass in CS treatment was mainly due to the increase in the amount of G^−^ bacteria, which increased by 106%. The application of CS increased the F/B ratio but reduced the G^+^/G^−^ ratio (Figure 3B). The number of CAZymes and gene families identified in the entire metagenome was 180,463 and 491, respectively. Among these CAZymes and gene families, bacteria and fungi accounted for 97.9 and 0.05%, respectively. The ACE indexes of bacterial and fungal communities were 11.4 and 27.1% higher in CF treatment than those in CS treatment, respectively (*p* < 0.05, Supplementary Figure S2A), while the Shannon index showed no difference (*p* > 0.05, Supplementary Figure S2B). PCoA demonstrated that the compositions of soil bacterial and fungal community were clearly distinguished under different fertilizer addition with 50.12 and 42.75% explanations of the first axis, respectively (*p* < 0.001, Figures 3C,D). CS application considerably changed soil bacterial and fungal community compositions compared to the CF treatment. The dominant phyla for bacteria were Pseudomonadota, Actinomycetota, and Acidobacteriota. The relative abundance of Actinomycetota was the highest in CF treatments, while that of Pseudomonadota became the highest in CS treatment. In addition, microbial biomarker species were noted to be more abundant in CS treatment (LDA > 3, *p* < 0.05, Figure 3E). Vicinamibacteraceae, Steroidobacteraceae, Lysobacteraceae and Ilumatobacteraceae were identified as biomarkers at the family level in CS treatment, exhibiting the increase by 10.6, 1.30, 22.5 and 12.5 fold compared with CF treatment, respectively. Ascomycota was the dominant fungal phylum, exhibiting a further increase in relative abundance in the CS treatment (Supplementary Figure S2D). While no significant difference was found for fungal biomarkers at the phylum level, primary enrichments were noted within family groups, including Podosporaceae and Nectriaceae (Figure 3F).

### 3.5 CAZymes related to the decomposition of plant and microbial necromass

**Figure 4:**
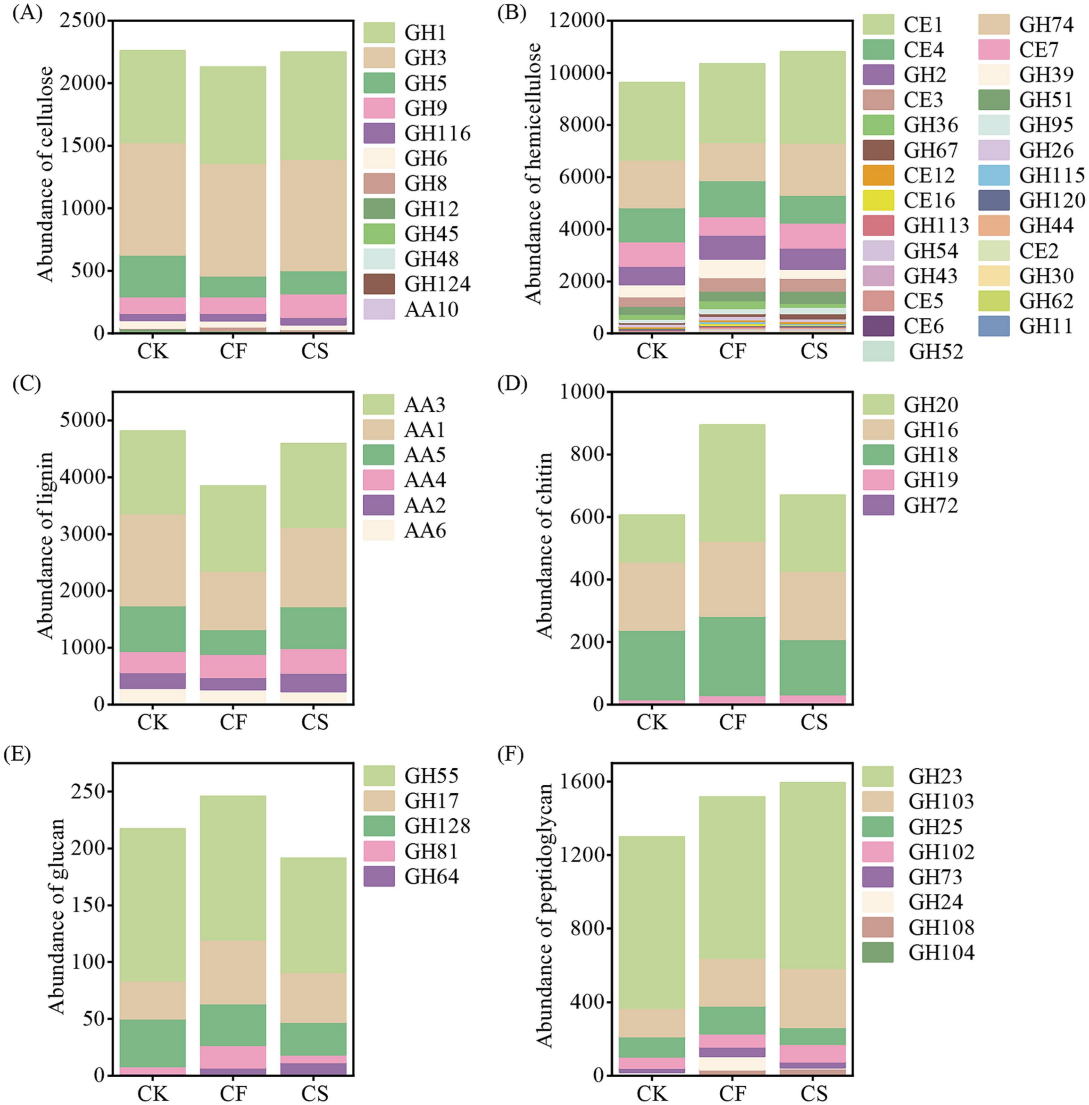
Abundance of specific CAZymes encoding the decomposition of plant-derived and microbial-derived components. Cellulose **(A)**, hemicellulose **(B)** and lignin **(C)** were components derived from plants. Chitin **(D)** and glucan **(E)** were components derived from fungus and peptidoglycan **(F)** was a component derived from bacteria.

The abundance of CAZymes related to the breakdown of plant-derived compounds was higher than that related to microbial-derived compounds decomposition (Figures 4A–F). Among plant-derived components, the abundance of CAZyme families encoding breakdown of hemicellulose surpassed those encoding cellulose and lignin decomposition. The highest abundance of gene families associated with the breakdown of plant residues were CE1 and GH74, both of which showed a significant enhancement with CS application (*p* < 0.05, Figure 4B). The most abundant CAZymes encoding fungal necromass breakdown was GH20 in CF and CS treatments and GH18 in CK treatment (Figure 4D), and the dominant CAZyme responsible for the breakdown of bacterial necromass was GH23 (Figure 4F).

The specific CAZymes encoding the breakdown of plant and microbial residue fractions varied in different treatments. The CAZymes associated with plant-and bacterial-derived compounds decomposition increased, whereas those encoding fungal-derived components decomposition reduced in CS treatment compared with CF treatment. The abundance of gene families CE1, GH74, and CE7 related to hemicellulose degradation increased by 468, 536, and 253 RPKM, respectively (Figure 4B). The abundance of gene families AA1 and AA5 encoding the breakdown of lignin was lower in CF treatment than in CS treatment (Figure 4C). GH3 and GH1 were the main gene families for cellulose degradation, and no significant difference in abundance was observed between CS and CF treatments. For the CAZymes responsible for decomposition of microbial residue fractions, the abundance values of GH20 and GH18, which are responsible for chitin degradation, were 128 and 76.9 RPKM lower in CS treatment than in CF treatment, respectively (Figure 4D). A similar trend was noted for the abundance values of GH24 and GH25 gene families related to peptidoglycan decomposition, with reductions of 66.3 and 59.0 RPKM, respectively, while the abundance values of the GH23 and GH103 gene families increased by 131 and 63.4 RPKM, respectively (Figure 4F).

**Figure 5:**
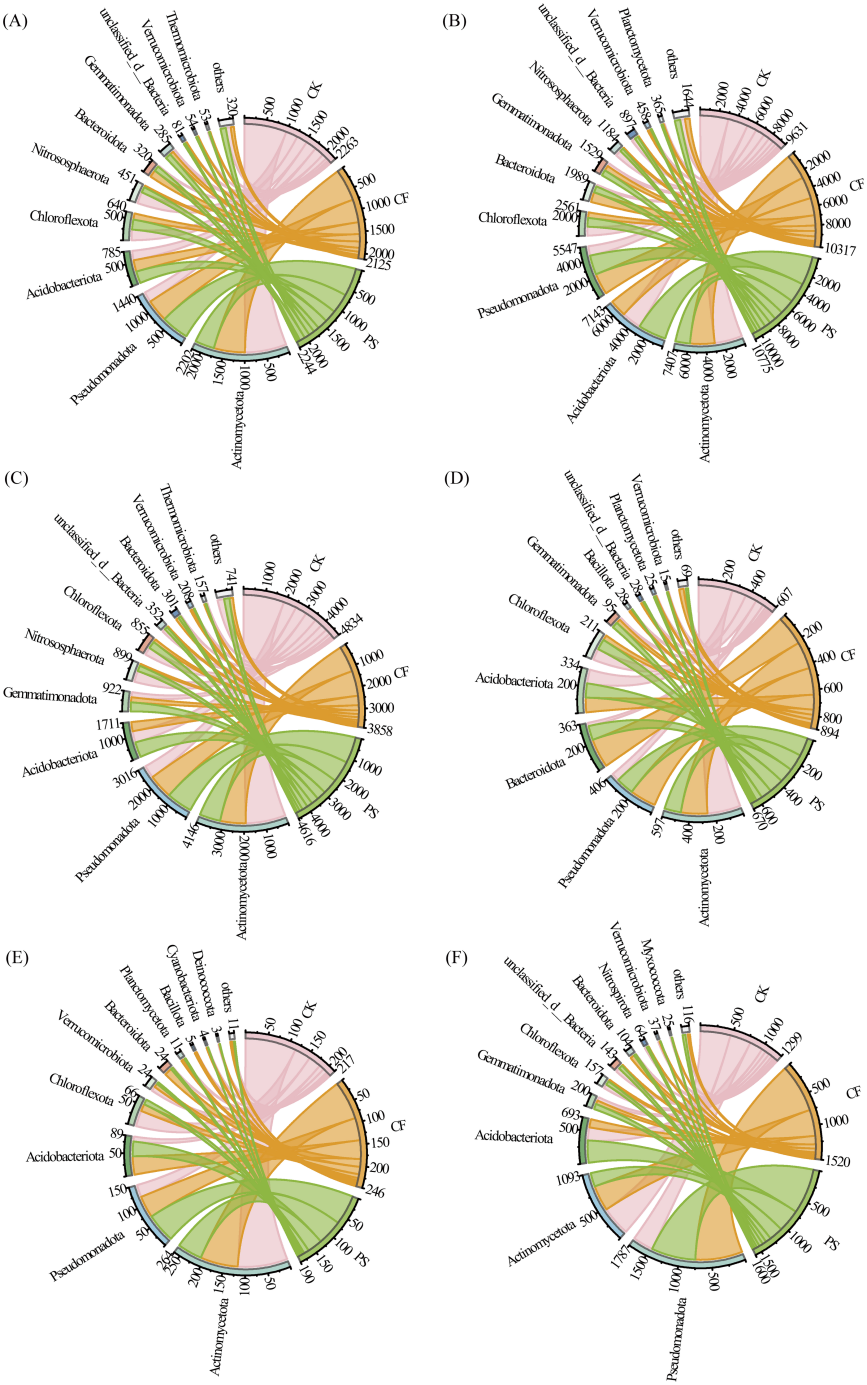
Contribution of microbial (bacterial and fungal) phyla to CAZyme genes for the decomposition of plant-and microbial-derived components. Cellulose **(A)**, hemicellulose **(B)** and lignin **(C)** were components derived from plants. Chitin **(D)** and glucan **(E)** were components derived from fungus and peptidoglycan **(F)** was a component derived from bacteria.

The CAZymes encoding the plant and microbial necromass decomposition were primarily attributed to the bacterial communities, with Acidobacteriota, Pseudomonadota, and Actinomycetota being the dominant phyla (Figures 5A–F). The abundance of species associated with the breakdown of fungal-derived peptidoglycan and plant-derived hemicellulose increased in CF and CS treatments compared with CK (*p* < 0.05, Figures 5B,F). CS application enhanced the abundance of species responsible for cellulose, hemicellulose, lignin, and peptidoglycan decomposition and reduced that for chitin and glucan degradation compared with CF application. The abundance of cellulose-degrading species in CS application increased owing to Pseudomonadota, Nitrososphaerota, and Chloroflexota (Figure 5A). Acidobacteriota and Chloroflexota mainly contributed to the increase of hemicellulose, lignin, and peptidoglycan degradation (Figure 5B). The abundance Actinomycetota and Bacteroidota for each residue component decomposition showed a similar decreasing trend in CS treatment compared with CF treatment.

### 3.6 Relation between soil environmental factors, specific CAZyme genes, and SOC accumulation

**Figure 6:**
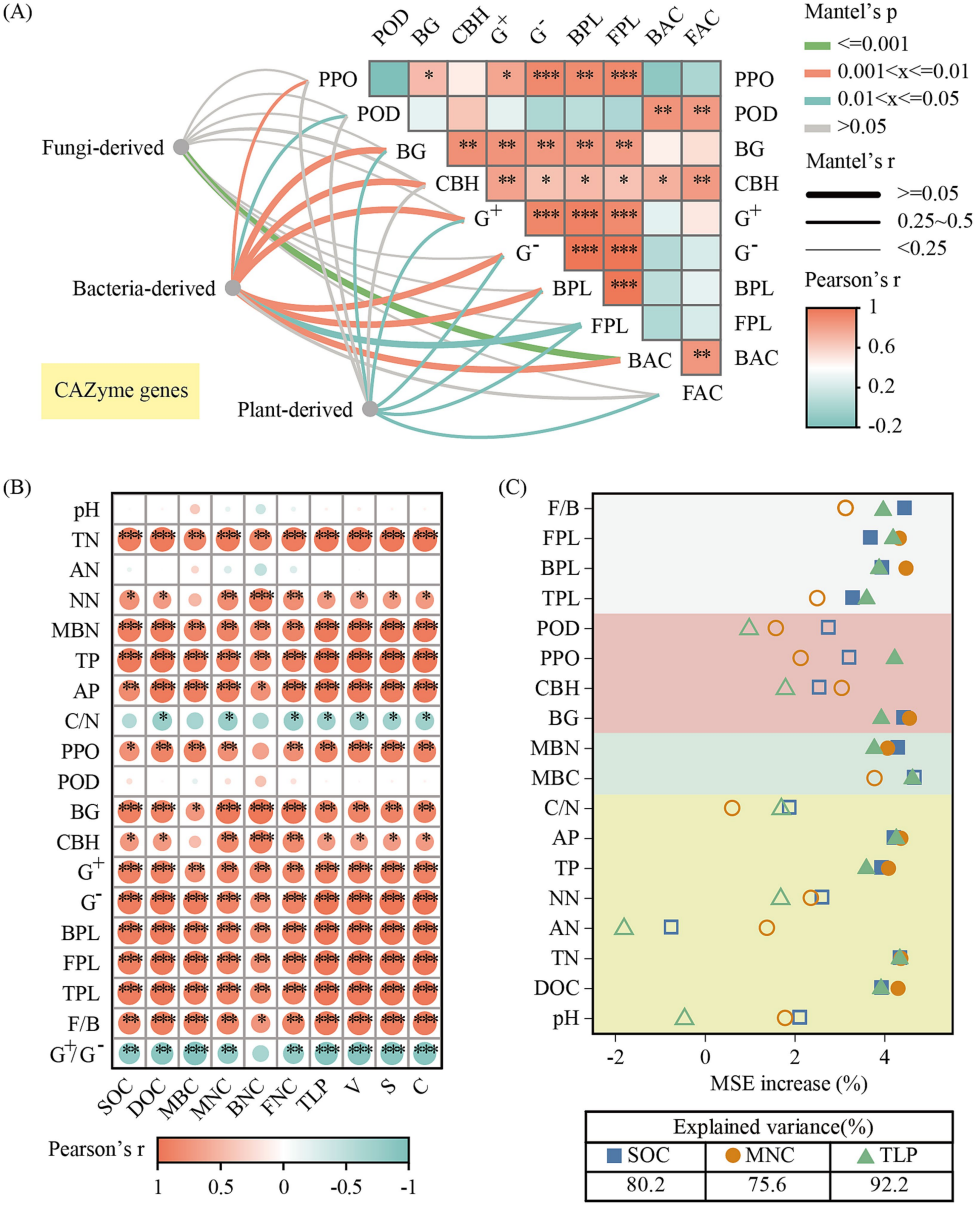
Mantel test analysis between the abundance of the CAZyme genes encoding the decomposition of plant- derived (cellulose, hemicellulose and lignin) and microbial-derived (chitin, glucan and peptidoglycan) components and soil enzyme activities **(A)**. Mantel’s p and r values are represented by the color and width of the connection line specified in the legend. Pearson correlation analysis between soil physical–chemical properties, soil enzyme activity, soil microbial biomass and the contents of SOC fractions **(B)**. Relative importance of independent variables controlling SOC, MNC and LP as represented by the percentage increase of the mean squared error (MSE%) based on random forest model **(C)**. The significant factors (*p* < 0.05) are indicated as solid colored symbols. BPL, bacterial PLFAs; FPL, fungal PLFAs; BAC, ACE index of bacterial communities; FAC, ACE index of fungal communities.

Soil enzyme activities had a positive and strong association with bacterial and fungal biomass, with the exception of POD activity (*p* < 0.05, Figure 6A). CAZymes responsible for the breakdown of bacterial-derived components was strongly correlated with soil enzyme activities and microbial biomass. There was a close relation between BG activity and the CAZymes responsible for the plant-derived fractions degradation. However, the abundance of CAZymes responsible for fungal-derived component degradation demonstrated no correlation with any enzyme activity (Figure 6A).

Pearson correlation analysis revealed that the contents of SOC and SOC fractions increased with the increasing TN, TP, and AP contents. Additionally, MNC and TLP were positively correlated with PPO, BG, and CBH activities but showed no significant correlation with POD activity (Figure 6B). Based on the random forest model, these variables explained 80.2, 75.6, and 92.2% of the total variance for SOC, MNC, and TLP, respectively (Figure 6C). DOC, TN, AP, MBN, BG, BPL, and FPL were the important factors of SOC, MNC, and TLP contents, suggesting that nutrients might be acquired by microbes to promote C accrual. The F/B ratio was the main predictor of MNC and TLP contents.

## 4 Discussion

### 4.1 Long-term application of CS had no impact on the contribution of MNC to SOC

In agricultural ecosystems, microbial necromass is essential for long-term conservation and sequestration of SOC and plays a vital role in SOC accumulation (Jia et al., 2017; Zhou et al., 2023). The application of CF and CS increased the MNC contents, including BNC and FNC in our study (Figure 1A), which was in line with the first hypothesis. Fertilizers might promote root biomass inputs and microbial proliferation, as well as increase N availability to enhance microbial C use efficiency, thereby promoting microbial necromass enrichment (Chen J. G. et al., 2020; Hu et al., 2022). The use of CS further enhanced the MNC content in comparison with the utilization of CF, possibly due to the following three reasons. Firstly, CS provided abundant bioavailable organic matter and nutrients, which was beneficial for microbial growth, resulting in larger microbial biomass (Fox et al., 2017; Zhou et al., 2023). Secondly, CS application supplied abundant nutrient substrates and was conducive to microbial proliferation (Table 1 and Figure 3A), thereby facilitating enrichment of microbial necromass (Chen et al., 2024; Yang et al., 2022; Ye et al., 2019). MNCs were positively correlated with PLFAs in this study, verifying that the increase in microbial biomass promoted microbial residue accrual (Supplementary Figure S3). Finally, the microorganisms and microbial residues in CS and soil are sources of amino sugars; thus, CS application directly and indirectly promotes long-term MNC accumulation (Chen X. B. et al., 2020; Jost et al., 2011). However, the contribution of MNC to SOC showed no difference among treatments (Figure 1B), indicating that CS application did not enhance the proportion of MNC to SOC, which contradicted our hypothesis. This might be explained by the simultaneous increase in MNC, combined with the dilution effect of increased plant necromass contribution to SOC, leading to the MNC contribution unchanged.

The content and proportion of FNC in MNC were far higher than those of BNC in our study (Figure 1A), demonstrating that FNC was dominant over BNC for MNC formation. The result may be partly explained by the fact that fungal necromass exhibit greater recalcitrance than bacterial necromass. The bacterial cell wall primarily comprises relatively labile components such as peptidoglycan, whereas the fungal cell wall primarily comprises less decomposable components such as chitin (Chen X. B. et al., 2020; Li et al., 2015). This stability of components facilitated the accumulation of fungal necromass, although fungi having lower biomass than bacteria in this study. In addition, the strong sorption of fungal hyphae in soil minerals could protect it from decomposition by soil enzymes and increase fungal necromass retention (Joergensen, 2018; Luo et al., 2022).

### 4.2 Long-term application of CS increased lignin phenols content

Accumulation of plant-derived C may be determined by the balance between the input and decomposition of plant-derived necromass (Wang et al., 2022). TLP content significantly increased under fertilization (Figure 2A), which might be explained by the enhancement of lignin input with the increased root biomass and unfavorable habitats for lignin major decomposers in croplands such as white-rot fungi (Su et al., 2023). Compared with CF treatment, the content and contribution to SOC of lignin phenols in CS treatment remarkably increased, as well as V-, S-, and C-type phenols (Figures 2A,B). CS application might facilitate lignin preservation but suppress lignin degradation. Greater S/V and C/V ratios indicate less microbial transformation, while the enhancement in the ratios of (Ad/Al)v and (Ad/Al)s show more side chain oxidation (Ma et al., 2018; Yang et al., 2022). Increased C/V and decreased (Ad/Al)v in CS treatment showed reduced lignin degradation in our study (Figures 2C,D). CS application enhanced lignin decomposition with higher plant inputs but less degradation of preserved lignin in the soil, thus increasing the selective retention of plant lignin. In addition, CS supplied high-available and preferred C sources for microorganisms, thereby reducing the attack from microbes and preserving more lignin (Li et al., 2020). Therefore, the organic components from CS application increased the lignin retention and accrual in soil.

SOC is generally comprised of microbial- and plant-derived C (Huang et al., 2022; Whalen et al., 2022). The unchanged contribution of MNC to SOC suggested that there might be no alteration in the contribution of plant-derived C as expected. Meng et al. (2024) found that despite the positive relationship of lignin phenols with SOC, they are not suitable as the sole biomarker for plant residues alone. Therefore, some other sources of plant necromass should be considered when evaluating the contribution of plant-derived C to SOC.

### 4.3 Long-term application of CS altered the microbial community and C-degrading genes

The application of CF and CS resulted in a notable separation of microbial community structure and differences in microbial community composition (Figures 3C–F and Supplementary Figure S2). CS application significantly promoted G^−^ bacteria growth and decreased the G^+^/G^−^ ratio. As biomass varied, the dominant bacterial phyla shifted from Actinomycetota (G^+^ bacteria) to Pseudomonadota (G^−^ bacteria) from CF treatment to CS treatment, with a notable enhancement of Acidobacteriota (G^−^ bacteria). Pseudomonadota are rapid-growing eutrophs that thrive in C-rich habitats and proficient in decomposing labile plant necromass C, such as cellulose and lignin (Fierer et al., 2007; Wang et al., 2023; Wiseschart et al., 2019). Acidobacteriota can produce various extracellular enzymes and possess an outstanding metabolic capacity to break down organic compounds (Richy et al., 2024; Zifcáková et al., 2016). For fungi, Ascomycota was the dominant phylum, and CS application remarkably enhanced its relative abundance. Ascomycota are effective in the breakdown of plant-derived cellulose and hemicellulose due to their abundant enzyme pool (Li et al., 2022; Manici et al., 2024). These variations in microbial communities indicated CS application accelerated plant residue conversion to microbial necromass.

Changes in microbial communities could drive shifts in microbial ecological functions, such as organic substrates degradation capacity and preference (Cheng et al., 2017). CAZyme genes assignment for degrading complex and simple C sources varied across treatments. The abundance of CAZyme genes encoding the breakdown of plant necromass was larger in CS than that in CF. The abundance of gene families CE1, GH47, and CE7 encoding for hemicellulose degradation (Figure 4B) and gene families AA1 and AA5 encoding for lignin degradation increased (Figure 4C), suggesting higher plant-derived necromass degradation into small C fractions. This might be due to the fact that the production of C-degrading enzymes is to a large extent dependent on energy substance adequacy (Chen Q. L. et al., 2020). A stronger ability of microorganisms to break down hemicellulose and lignin for obtaining more C resources corresponded with increased the biosynthesis of CAZyme genes (Huang et al., 2024; Trivedi et al., 2016). For microbial-derived fractions, the gene families GH16, GH17, GH23, and GH103 confirmed their functions in microbial necromass decomposition (Zifcáková et al., 2017). The elevated abundance of GH23 and GH103, which are associated with peptidoglycan decomposition (Figure 4F), suggests a higher potential for bacterial necromass degradation. In addition, the pool of CAZyme genes encoding breakdown of plant and microbial necromass was distinct, with higher abundance of CAZymes for plant necromass breakdown than that for microbial necromass breakdown. These results indicated a larger investment of plant necromass for C pool and corresponded with the previous viewpoint that plant necromass is rich in organic substance and most likely constitutes a substantial portion of soil C sink (Ren C. J. et al., 2021; Zhang B. G. et al., 2021).

### 4.4 Mechanism of long-term application of CS affecting SOC accumulation

**Figure 7:**
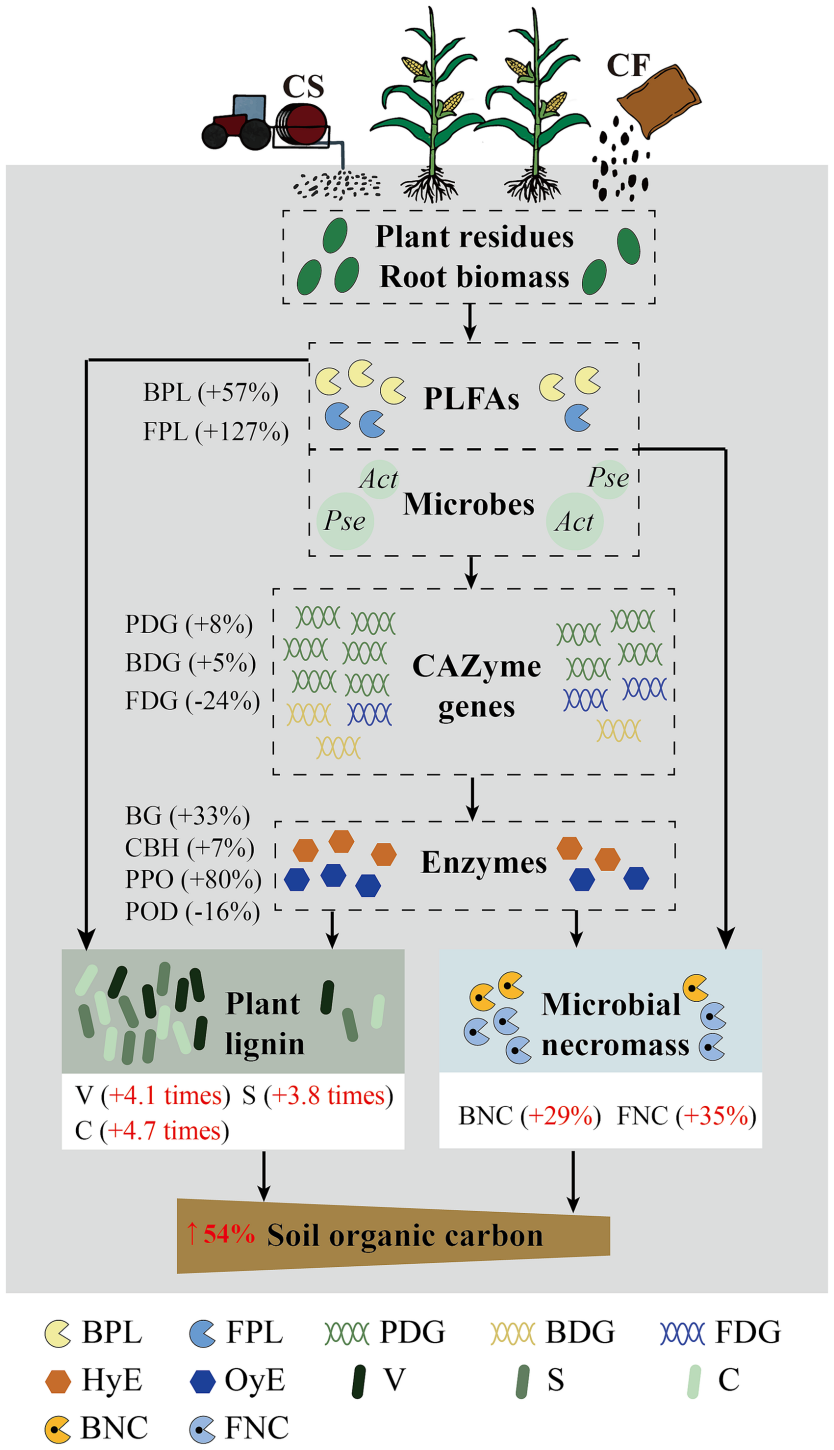
A conceptual figure of the influence of cattle slurry (CS) and chemical fertilizer (CF) application on SOC formation. The numerical labels indicates the effect of CS for each indicator compared with CF. HyE, hydrolase (BG and CBH); OyE, oxidase (PPO and POD); PDG, CAZyme genes encoding the decomposition of plant-derived components; BDG, CAZyme genes encoding the decomposition of bacterial-derived components; FDG, CAZyme genes encoding the decomposition of fungal-derived components.

The contents of SOC, MNC, and TLP increased from the application of CF to CS. Substrate availability, microbial communities, and enzyme activities are significant factors leading to the SOC enhancement (Figure 6C). The probable mechanism by which CS application improved SOC accumulation is depicted in Figure 7. CS application may boost plant residue input and exogenous C sources, increasing soil nutrients and DOC content. Higher DOC content might promote microbial biomass synthesis by improving microbial metabolism, which was conducive to MNC enrichment (Zifcáková et al., 2017). Furthermore, the shifts of dominant microbial phylum from oligotrophic to eutrophic reflected the alterations of microbial community functions (Domeignoz-Horta et al., 2021). Microbial community features were vital factors affecting MNC and TLP. The correlation between microbial community features and functional CAZyme genes explained the regulative role of soil microorganisms in soil C cycling and the vital role of dominant CAZyme families in C source turnover. Changes in CAZyme genes encoding plant and microbial necromass decomposition would affect microbial metabolism via a “microbial carbon pump,” thereby facilitating SOC formation and accumulation. The CK treatment in this study involved abandoned land restored from former maize cultivation without fertilization, rather than maize fields without fertilizer application. This limitation of our work might have introduced variability in SOC conditions, although the primary focus of this study was on the relative differences between CS and CF treatments. Future studies should address the limitation and aim to include a strict maize-without-fertilizer control to further isolate fertilization effects on SOC dynamics. Although this CK treatment differed from the conventional definition of a control, it provided a valuable baseline for assessing SOC dynamics under conditions of minimal anthropogenic inputs. This study offered valuable insights into the underlying connection between SOC degradation and microbial CAZyme genes under the long-term application of cattle slurry.

## 5 Conclusion

Long-term application of CS substantially enhanced SOC accumulation compared with CF treatment, primarily through increased microbial- and plant-derived C. CS application promoted plant biomass input and reduced the oxidative degradation of lignin, leading to a higher lignin phenol content in soil. Although CS stimulated microbial biomass production and MNC accumulation, the contribution of MNC to total SOC remained stable. Compared to CF, CS application induced shifts in microbial community composition, favoring eutrophic bacterial groups such as Pseudomonadota, and enhanced the abundance of CAZyme genes involved in plant and bacterial biomass degradation. These changes suggest a greater potential for substrate turnover and carbon stabilization under CS management. Overall, the availability of substrates, microbial community, and enzyme activities are significant factors attributing to the increase of SOC contents. Our findings offered support for the underlying connection between SOC degradation and microbial CAZyme genes and provide important scientific evidence to support the sustainable recycling of livestock manure in cropland systems.

## Data Availability

The datasets presented in this study can be found in online repositories. The names of the repository/repositories and accession number(s) can be found in the article/Supplementary material.

## Footnotes

^1^ https://www.ncbi.nlm.nih.gov/

^2^ http://www.CAZy.org
