# Supplementary material for: Divergent responses of plant lignin and microbial necromass to the contribution of soil organic carbon under organic and chemical fertilization
Source: Front Microbiol. 2025 May 16;16:1586791. doi: 10.3389/fmicb.2025.1586791 (PMC12124130; doi:10.3389/fmicb.2025.1586791)
Supplement: Supplementary file 1 [file Table_1.docx]

Supplementary Material

# Supplementary Materials and Methods

**Soil physical and chemical properties analysis.**

The soil total nitrogen (TN) was determined using the Kjeldahl method. The soil nitrate nitrogen (NN) and ammonium nitrogen (AN) were measured via a continuous flow analyser (Autoanalyzer 3, Bran and Luebbe, Germany). The soil total phosphorus (TP) and available phosphorus (AP) concentrations were assayed by melt-molybdenum, antimony and scandium colorimetry (Bao, 2000). The soil pH was measured with a soil pH meter according to a water/soil ratio of 2.5:1(v/w). Enzyme activities, including POD, PPO, BG and CBH, were assessed using the G0317W, G0311W, G0312W96 and G0323W96 assay kits (Suzhou Grace Biotechnology Co., Ltd., China) according to the manufacturer’s instructions.

Bao, S.D. (2000). Soil and Agricultural Chemistry Analysis. China Agriculture Press, Beijing, China (in Chinese)

# Supplementary Figures and Tables

## Supplementary Figures


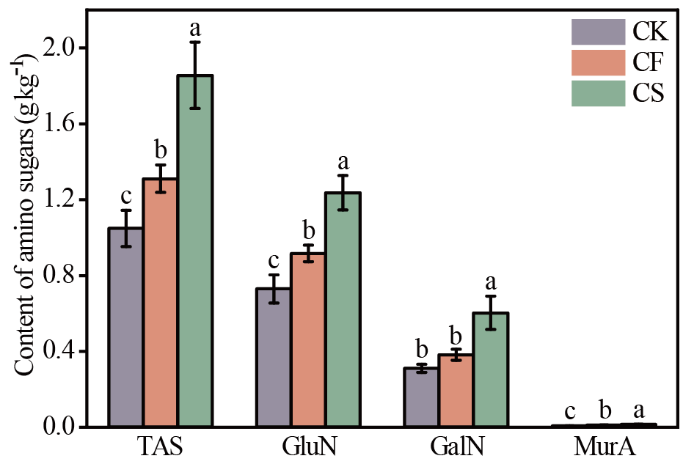


**Fig. S1.** Contents of total amino sugars, glucosamine, galactosamine and muramic acid in soil under different treatments. Error bars represent standard errors of the means (n = 3). Different letters indicate significant differences among the treatments (*p* < 0.05, Duncan’s test).


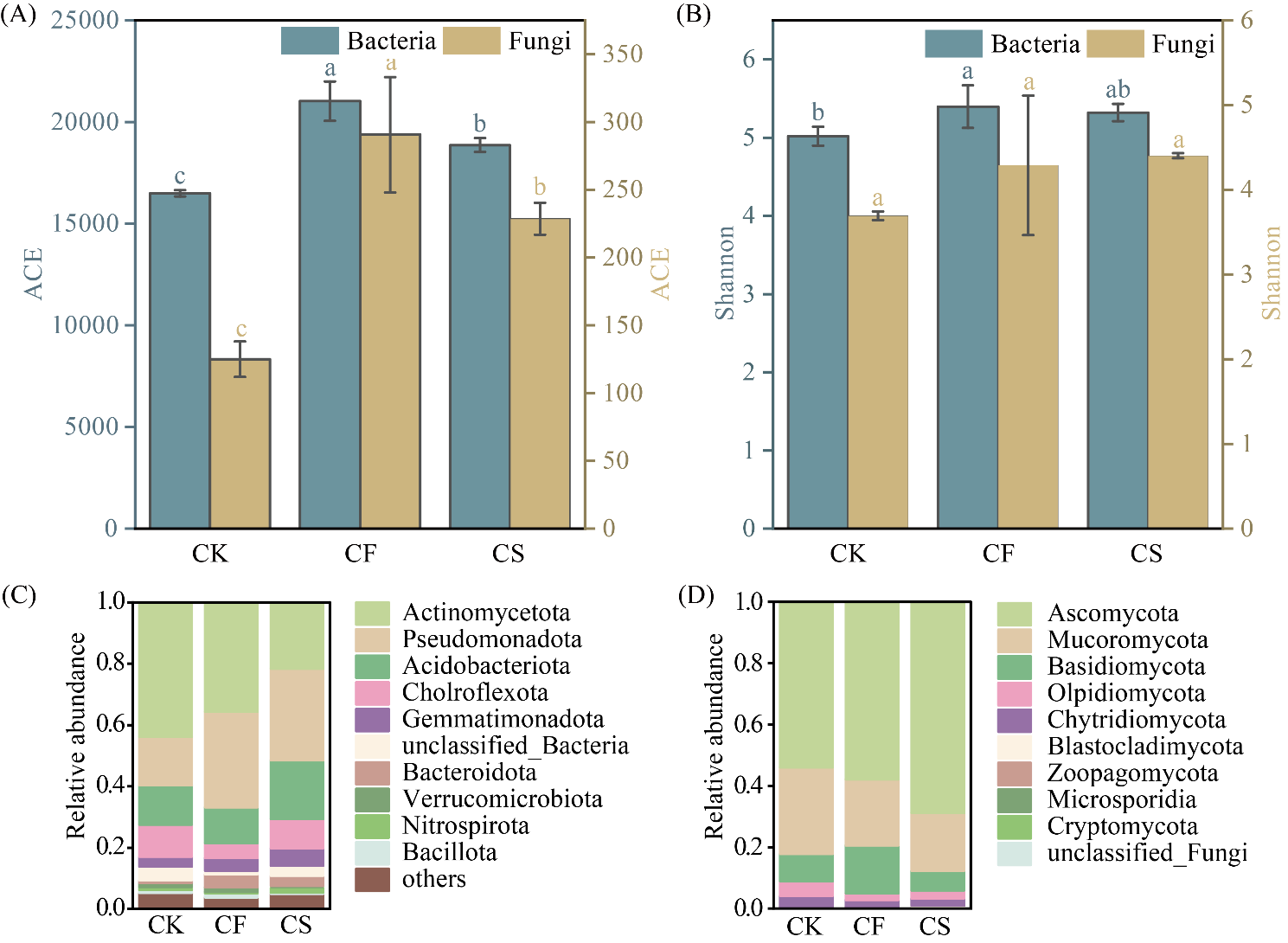


**Fig. S2.** Diversity indices of bacterial **(A)** and fungal communities **(B)**. Relative abundance of top ten bacteria **(C)** and fungi **(D)** at phylum level in soil under different treatments. Different letters indicate significant differences in the diversity indices of bacterial and fungal communities among the treatments (*p* < 0.05, Duncan’s test).


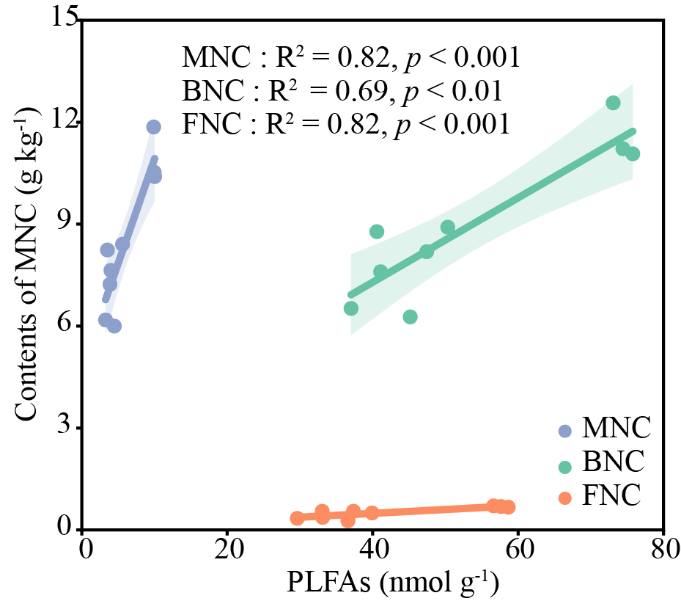


**Fig. S3.** Corrections of MNC contents and PLFAs. MNC: microbial necromass C; FNC: fungal necromass C; BNC: bacterial necromass C.

## Supplementary Tables

**Table S1.** Phospholipid fatty acid (PLFA) biomarkers chosen to characterize microbial community composition.

| Microbial group | Fatty acid biomarkers | Reference |
| --- | --- | --- |
| Gram-positive bacteria | 14:0 anteiso, 14:0 iso, 15:0 anteiso, 15:0 iso, 15:1 iso w6c, 16:0 anteiso, 16:0 iso, 17:0 anteiso, 17:0 iso, 17:1 iso w9c, 19:0 anteiso | Cui et al. (2021); Joergensen (2022) |
| Gram-negative bacteria | 14:0 iso 3OH, 14:1 w8c,15:1 w7c, 16:0 2OH, 16:1 w7c, 16:1 w7c DMA,  16:1 w9c, 17:0 cyclo w7c, 17:0 iso 3OH, 17:1 w8c, 18:1 w5c, 18:1 w7c, 19:0 cyclo w7c, 20:1 w9c | Shen et al. (2018) |
| Unspecified bacteria | 14:00, 15:00, 16:00, 17:00, 18:00, 20:00 | Yang et al. (2017); Wang et al. (2018) |
| Fungi | 16:1 w5c, 18:1 w9c, 18:2 w6c | Willers et al. (2015) |

Cui, J.W., Song, D.L., Dai, X.L., Xu, X.P., He, P., Wang, X.Y., Liang, G.Q., Zhou, W., Zhu, P. (2021). Effects of long-term cropping regimes on SOC stability, soil microbial community and enzyme activities in the Mollisol region of Northeast China. Applied Soil Ecology 164, 13. doi: 10.1016/j.apsoil.2021.103941

Joergensen, R.G. (2022). Phospholipid fatty acids in soil-drawbacks and future prospects. Biology and Fertility of Soils 58, 1-6. doi: 10.1007/s00374-021-01613-w

Shen, D.Y., Ye, C.L., Hu, Z.K., Chen, X.Y., Guo, H., Li, J.Y., Du, G.Z., Adl, S., Liu, M.Q. (2018). Increased chemical stability but decreased physical protection of soil organic carbon in response to nutrient amendment in a Tibetan alpine meadow. Soil Biology & Biochemistry 126, 11-21. doi: 10.1016/j.soilbio.2018.08.008

Wang, C., Lu, X.K., Mori, T., Mao, Q.G., Zhou, K.J., Zhou, G.Y., Nie, Y.X., Mo, J.M. (2018). Responses of soil microbial community to continuous experimental nitrogen additions for 13 years in a nitrogen-rich tropical forest. Soil Biology & Biochemistry 121, 103-112. doi: 10.1016/j.soilbio.2018.03.009

Willers, C., van Rensburg, P.J.J., Claassens, S. (2015). Phospholipid fatty acid profiling of microbial communities-a review of interpretations and recent applications. Journal of Applied Microbiology 119, 1207-1218. doi: 10.1111/jam.12902

Yang, S., Xu, Z.W., Wang, R.Z., Zhang, Y.Y., Yao, F., Zhang, Y.G., Turco, R.F., Jiang, Y., Zou, H.T., Li, H. (2017). Variations in soil microbial community composition and enzymatic activities in response to increased N deposition and precipitation in Inner Mongolian grassland. Applied Soil Ecology 119, 275-285. doi: 10.1016/j.apsoil.2017.06.041

**Table S2.** The detailed information of CAZymes families involved in the degradation of plant-derived (cellulose, hemicellulose and lignin) and microbial-derived (chitin, glucan and peptidoglycan) components (<http://www.CAZy.org>).

| Components | Description | CAZymes families |
| --- | --- | --- |
| Cellulose | β-glucosidase (EC 3.2.1.21) | GH1, GH3, GH116 |
|  | cellulose β-1,4-cellobiosidase (EC 3.2.1.91) | GH5 |
|  | reducing end-acting cellobiohydrolase (EC 3.2.1.176) | GH48 |
|  | endo-β-1,4-glucanase (EC 3.2.1.4) | GH124, GH45, GH6, GH9, GH12 |
|  | cellulase (EC 3.2.1.4) | GH8 |
|  | Lytic chitin monooxygenase (EC 1.14.99.53) | AA10 |
| Hemicellulose | acetyl xylan esterase (EC 3.1.1.72) | CE1, CE2, CE3, CE4, CE5, CE6, CE7, CE12, CE16 |
|  | α-L-arabinofuranosidase (EC 3.2.1.55) | GH62, GH51, GH54 |
|  | β-mannanase (EC 3.2.1.78) | GH113, GH26 |
|  | endo-β-1,4-xylanase (EC 3.2.1.8) | GH11, GH30 |
|  | β-xylosidase (EC 3.2.1.37) | GH120, GH52, GH39, GH43 |
|  | xylan α-1,2-glucuronidase (EC 3.2.1.131) | GH115 |
|  | α-glucuronidase (EC 3.2.1.139) | GH67 |
|  | xyloglucanase (EC 3.2.1.151) | GH44, GH74 |
|  | β-galactosidase/β-glucuronidase (EC 3.2.1.-) | GH2 |
|  | α-galactosidase (EC 3.2.1.22) | GH36 |
|  | α-L-fucosidase (EC 3.2.1.51) | GH95 |
| Lignin | Laccase (EC 1.10.3.2) | AA1 |
|  | manganese peroxidase (EC 1.11.1.13) | AA2 |
|  | Oxidase (EC 1.1.3.-) | AA3, AA4, AA5 |
|  | P-benzoquinone reductase (NADPH) (EC 1.6.5.6) | AA6 |
| Chitin | endo-xyloglucanase (EC 3.2.1.151) | GH16 |
|  | chitinase (EC 3.2.1.14) | GH18, GH19 |

**Continued Table S2.**

| Components | Description | CAZymes families |
| --- | --- | --- |
| Chitin | N-acetyl β-glucosaminidase (EC 3.2.1.-) | GH20 |
|  | β-1,3-glucanosyltransglycosylase (EC 2.4.1.-) | GH72 |
| Glucan | β-1,3-glucanase (EC 3.2.1.39) | GH64, GH128 |
|  | glucan endo-1,3-β-glucosidase (EC 3.2.1.39) | GH17 |
|  | exo-β-1,3-glucanase (EC 3.2.1.58) | GH55 |
|  | endo-β-1,3-glucanase (EC 3.2.1.39) | GH81 |
| peptidoglycan | lysozyme type G (EC 3.2.1.17) | GH23 |
|  | lysozyme (EC 3.2.1.17) | GH24, GH25, GH73 |
|  | peptidoglycan lytic transglycosylase (EC 3.2.1.-) | GH102, GH103, GH104 |
|  | N-acetylmuramidase (EC 3.2.1.17) | GH108 |
